# Multi-Level Profiling of MAPK-Associated Genes and MicroRNAs Uncovers Regulatory Networks in Breast Cancer Subtypes

**DOI:** 10.3390/ijms262411831

**Published:** 2025-12-07

**Authors:** Katarzyna Król-Jatręga, Elżbieta Mitka-Krysiak, Kacper Boroń, Piotr Ossowski, Nikola Zmarzły, Paweł Ordon, Wojciech Kulej, Tomasz Sirek, Agata Sirek, Dariusz Boroń, Grzegorz Wyrobiec, Yuriy Prudnikov, Beniamin Oskar Grabarek

**Affiliations:** 1 Collegium Medicum, WSB University, 41-300 Dabrowa Gornicza, Poland; elzbietamitkakrysiak@gmail.com (E.M.-K.); drpiotrossowski@gmail.com (P.O.); nikola.zmarzly@gmail.com (N.Z.); wojciechkulej87@gmail.com (W.K.); drtstierka@gmail.com (T.S.); agatasirek06@gmail.com (A.S.); dariusz@boron.pl (D.B.); yuriy.prudnikov@wsb.edu.pl (Y.P.); bgrabarek7@gmail.com (B.O.G.); 2 Department of Plastic Surgery, Faculty of Medicine, Academia of Silesia, 40-555 Katowice, Poland; q375@icloud.com; 3 Department of Plastic and Reconstructive Surgery, Hospital for Minimally Invasive and Reconstructive Surgery in Bielsko-Biała, 43-316 Bielsko-Biala, Poland; 4 Faculty of Medicine and Health Sciences, Andrzej Frycz Modrzewski University in Kraków, 30-705 Kraków, Poland; 5 Department of Gynecology and Obstetrics, TOMMED Specjalisci od Zdrowia, 40-662 Katowice, Poland; 6 Department of Gynecology and Obstetrics with Gynecologic Oncology, Ludwik Rydygier Memorial Specialized Hospital, 31-826 Kraków, Poland; 7 Department of Histology and Cell Pathology in Zabrze, Faculty of Medical Sciences in Zabrze, Medical University of Silesia in Katowice, 41-808 Zabrze, Poland; gwyrobiec@sum.edu.pl

**Keywords:** breast neoplasms, MAP kinase signaling system, MicroRNAs, gene expression regulation

## Abstract

Breast cancer (BC) comprises heterogeneous subtypes with distinct molecular drivers and clinical behaviors. Among the key signaling pathways implicated in BC progression is the mitogen-activated protein kinase (MAPK) cascade, which regulates cell proliferation, apoptosis, and stress responses. microRNAs (miRNAs), as post-transcriptional regulators, are increasingly recognized as modulators of MAPK-associated genes, yet their integrated role across BC subtypes remains incompletely understood. This study included 405 patients with histopathologically confirmed BC, stratified into luminal A (LumA), HER2-negative luminal B, HER2-positive luminal B, non-luminal HER2-positive, and triple-negative breast cancer (TNBC). Control tissues were obtained from matched surgical margins. We performed mRNA profiling (Affymetrix microarrays), reverse transcription-quantitative polymerase chain reaction (RT-qPCR), protein quantification (enzyme-linked immunosorbent assay (ELISA), and miRNA expression analysis. Predicted miRNA-mRNA interactions were analyzed using the miRDB database. Functional protein–protein interactions were explored using the STRING database. MAP3K1, MAP2K4, and TP53 were significantly downregulated across all subtypes, while PPM1D, LMTK3, and TGFB1 were upregulated, especially in TNBC. These alterations were supported by concordant changes at the protein level. Dysregulated miRNAs—miR-21-3p, miR-23c, miR-27a-3p, miR-205-3p, and miR-300—exhibited in-verse expression patterns relative to their predicted target genes. STRING analysis identified TP53 as a central hub, linking MAPK signaling with stress and apoptotic pathways. This integrated transcriptomic and miRNA profiling study reveals subtype-specific dysregulation of MAPK-associated genes and their miRNA regulators in BC, with TNBC exhibiting the most profound alterations. These findings provide insight into potential targets for personalized therapeutic strategies.

## 1. Introduction

Breast cancer is not a single disease but a complex group of biologically distinct subtypes, each with its own molecular drivers, clinical behavior, and response to therapy [1,2]. Advances in genomic profiling have led to the classification of breast cancer into at least four major intrinsic subtypes: Luminal A, Luminal B, HER2-enriched, and triple-negative breast cancer (TNBC) [3,4,5]. Luminal A tumors, which are estrogen receptor (ER)-positive and low-proliferating, generally have the best prognosis [6]. They typically exhibit strong hormone sensitivity, low genomic instability, and limited activation of proliferative signaling pathways such as the mitogen-activated protein kinase (MAPK) cascade and the phosphoinositide 3-kinase/protein kinase B (PI3K/AKT) pathway, which contributes to their more favorable clinical course [7,8]. Luminal B represents a heterogeneous group characterized by estrogen-receptor positivity, higher proliferative activity, and a less favorable prognosis compared with Luminal A [5,9]. Importantly, Luminal B encompasses two biologically distinct subgroups that differ in their HER2 status [10]. The HER2-negative form is defined by ER positivity, variable progesterone receptor (PR) expression, a high Ki-67 index, and the absence of HER2 overexpression or amplification, whereas the HER2-positive form combines ER positivity with HER2 overexpression or amplification and typically demonstrates increased proliferative signaling [11,12,13]. These HER2-stratified Luminal B variants differ not only in clinical behavior and therapeutic responsiveness but also in their engagement of intracellular signaling cascades such as MAPK, PI3K/AKT, and transforming growth factor beta (TGF-β) pathways. Because these distinctions have direct implications for interpreting subtype-specific gene and miRNA alterations, the present study analyzes HER2-negative and HER2-positive Luminal B tumors as separate molecular entities [11,12,13]. In contrast, TNBC lacks expression of ER, PR, and HER2, and is associated with a poor prognosis due to limited treatment options and a high likelihood of recurrence [14,15,16,17,18].

Underlying these subtypes are diverse signaling pathways that contribute to tumor growth, invasion, and resistance. One of the central signaling networks involved in breast cancer pathogenesis is the mitogen-activated protein kinase (MAPK) pathway [19]. This highly conserved cascade integrates external signals through a series of kinases—including ERK1/2, JNK, and p38—that regulate key cellular processes such as proliferation, differentiation, survival, and inflammation [20,21]. Aberrant activation of MAPK signaling is commonly observed in breast cancer, often as a result of upstream mutations (e.g., RAS, BRAF), receptor overexpression (e.g., EGFR, HER2), or cross-talk with hormone receptor signaling [22].

Importantly, regulation of the MAPK pathway extends beyond protein-level signaling. microRNAs (miRNAs) [23]—short, non-coding RNAs that post-transcriptionally regulate gene expression [24]—have emerged as key modulators of MAPK-related genes [23]. Dysregulation of specific miRNAs can either amplify or suppress MAPK signaling outputs, depending on the cellular context and tumor subtype [25,26]. For example, some miRNAs directly target MAPK cascade components, while others influence upstream receptors or downstream effectors, collectively shaping tumor behavior and therapeutic responses [27,28]. In the present study, we focused on a restricted panel of miRNAs predicted to regulate MAPK-associated transcripts and dysregulated in our breast cancer cohort. In particular, miR-21-3p/5p has been repeatedly implicated as a pleiotropic oncomiR targeting key components of the PI3K/AKT, MAPK and TGF-β pathways, thereby promoting proliferation, survival, and chemoresistance [29]. miR-27a-3p is known to enhance tumor growth and invasion by repressing stress-responsive kinases and other tumor suppressors [30,31], whereas miR-205-3p has context-dependent roles, including regulation of EMT-related factors such as ZEB1/2 and PTEN, as well as components of immune and growth-factor signaling cascades [32,33]. Together with miR-23c and miR-300, which have been linked to MAPK and TP53 pathway modulation, these miRNAs constitute a functionally plausible regulatory layer capable of fine-tuning MAPK signaling outputs in a subtype-dependent manner [34].

MAPK signaling (ERK1/2, JNK, p38) integrates upstream cues from receptor tyrosine kinases, cytokine and GPCR signaling, and non-canonical TGF-β signaling. While numerous MAPK components have been implicated individually in breast cancer, there is limited, subtype-resolved work that jointly profiles MAPK transcripts, protein readouts, and candidate regulatory miRNAs within the same patient cohort to connect pathway activation with potential post-transcriptional control [20,35].

The current work represents the fourth in a coordinated series of analyses performed on a unified cohort of breast cancer patients, each focusing on distinct molecular signaling axes. Earlier studies characterized transcriptomic and miRNA alterations within the histaminergic, dopaminergic, dopaminergic, and transforming growth factor beta signaling pathway (SMAD/TGF-β) [36,37,38]. Building on these foundations, we now provide an integrative transcriptomics investigation of the MAPK signaling network and its potential post-transcriptional regulation by miRNAs across five intrinsic breast cancer subtypes. This approach allows cross-pathway comparison within the same patient population, revealing how MAPK activation may intersect with TGF-β, dopaminergic, and histaminergic signaling in subtype-specific contexts.

In this study, we aimed to bridge this gap by profiling the expression of MAPK-related mRNAs and their regulatory miRNAs in breast cancer, with an emphasis on differences between molecular subtypes.

## 2. Results

### 2.1. Differential Expression of MAPK-Associated Genes Across Breast Cancer Subtypes

Out of 300 MAPK pathway-associated genes screened, six genes showed statistically significant differential expression across all breast cancer subtypes when compared to control tissue (*p* < 0.05; |fold change| > 2), as summarized in Table 1. Among these, *MAP3K1*, *MAP2K4*, and *TP53* were consistently downregulated in tumor tissues across all subtypes, with log_2_fold-change values ranging from −2.87 to −5.71. Notably, *MAP3K1* and *TP53* showed the greatest downregulation in TNBC (−4.32 and −5.65, respectively). In contrast, *PPM1D*, *LMTK3*, and *TGFB1* were significantly upregulated across all subtypes, with the highest induction observed in TNBC (*PPM1D*: 4.87; *TGFB1*: 5.18). The upregulation of *TGFB1* and *LMTK3* was particularly marked in luminal subtypes and TNBC, suggesting potential subtype-specific regulatory roles.

**Figure 1:**
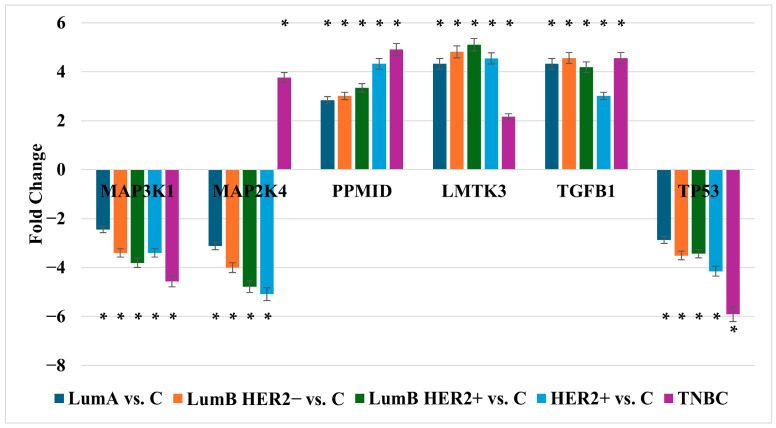
RTqPCR results. Data are presented as mean ± standard deviation (SD). * *p* < 0.05 vs. control.

These transcriptomic patterns were further validated by RT-qPCR analysis (Figure 1), which confirmed the direction and magnitude of gene expression changes detected by microarray profiling.

Bar plots represent mean fold change (±SEM) compared to control (C) tissue. LumA, luminal A; LumB, luminal B; HER2, human epidermal growth factor receptor 2; TNBC, triple-negative breast cancer; *MAP3K1*, mitogen-activated protein kinase kinase kinase 1; MAP2K4, mitogen-activated protein kinase kinase 4; *PPM1D*, protein phosphatase Mg^2+^/Mn^2+^ dependent 1D (WIP1); *LMTK3*, lemur tyrosine kinase 3; *TGFB1*, transforming growth factor beta 1; *TP53*, tumor protein p53.

### 2.2. External Validation of MAPK-Associated Gene Expression Using University of Alabama at Birmingham Cancer Data Analysis Portal (UALCAN)

**Figure 2:**
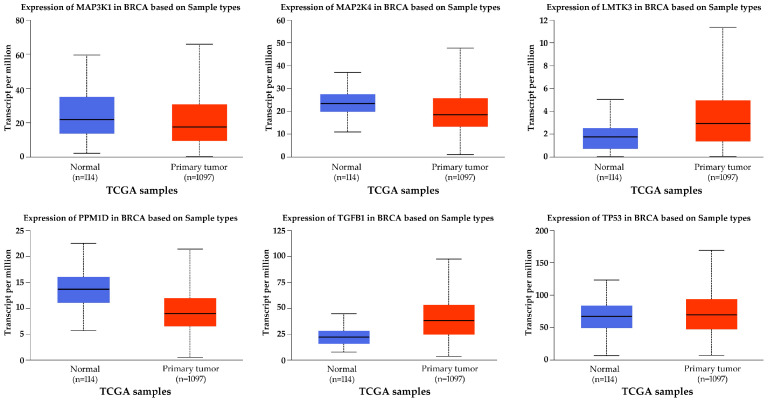
UALCAN Analysis of MAPK-Associated Gene Expression in Primary Breast Tumors vs. Normal Breast Tissue (TCGA-BRCA).

In the TCGA-BRCA dataset, four MAPK-related genes—*PPM1D*, *LMTK3*, *TGFB1*, and *TP53*—showed statistically significant differential expression between primary breast cancer samples and normal breast tissue (Figure 2; *p* < 0.05). *PPM1D*, *LMTK3*, and *TGFB1* were significantly upregulated in tumor samples relative to normal tissue, while *TP53* was significantly downregulated. For *MAP3K1* and *MAP2K4*, differences in expression between tumor and normal samples followed the same direction as in our microarray dataset but did not reach statistical significance in the UALCAN comparison (Figure 2).

**Figure 3:**
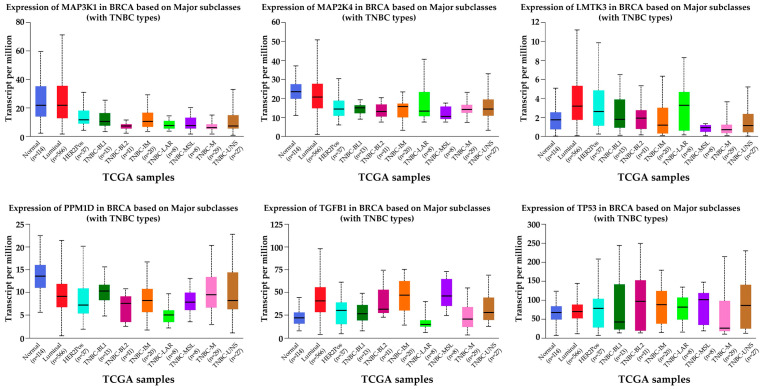
UALCAN Subtype-Resolved Expression of MAPK-Associated Genes Across Luminal, HER2-Positive, and TNBC Subclasses. TNBC-BL1, Triple-Negative Breast Cancer Basal-Like 1; TNBC-BL2, Triple-Negative Breast Cancer Basal-Like 2; TNBC-IM, Triple-Negative Breast Cancer Immunomodulatory; TNBC-M, Triple-Negative Breast Cancer Mesenchymal; TNBC-MSL, Triple-Negative Breast Cancer Mesenchymal Stem-Like; TNBC-LAR, Triple-Negative Breast Cancer Luminal Androgen Receptor; TNBC-UNS, Triple-Negative Breast Cancer Unspecified.

Subtype-level UALCAN analyses (Figure 3) demonstrated that MAPK-associated gene expression varied across luminal, HER2-positive, and triple-negative breast cancer groups. Luminal A and luminal B tumors exhibited moderate deviations from normal tissue for the analyzed genes. In HER2-positive tumors, more pronounced shifts were observed for *PPM1D*, *LMTK3*, and *TGFB1*. TNBC subclass analysis (TNBC-BL1, BL2, IM, M, MSL, LAR, UNS) revealed the broadest expression variability, with basal-like subclasses (BL1, BL2) showing the strongest reduction in *MAP3K1*, *MAP2K4*, and *TP53*, and mesenchymal-associated subclasses (M, MSL) demonstrating higher levels of *PPM1D*, *LMTK3*, and *TGFB1*.

### 2.3. Prediction of MAPK Pathway Genes Expression Regulation by miRNA

Five significantly dysregulated miRNAs were identified as potential regulators of MAPK pathway genes across all breast cancer subtypes (*p* < 0.05; |log_2_FC| > 2) (Table 2). miR-21-3p (↑) and miR-23c (↓) were predicted to target *MAP3K1*, showing inverse expression patterns. miR-27a-3p, targeting *MAP2K4*, was consistently upregulated, especially in HER2+ and TNBC subtypes. miR-205-3p, targeting *PPM1D*, was upregulated in luminal subtypes but downregulated in TNBC. miR-300, associated with *TP53* regulation, was significantly downregulated across all subtypes, most notably in TNBC. These patterns support subtype-specific post-transcriptional regulation of MAPK genes by miRNAs. While several miRNA–mRNA pairs demonstrated significant inverse correlations these data reflect computational predictions and correlative relationships rather than experimentally validated regulatory events.

### 2.4. Concentration of Selected Proteins in Breast Cancer Tissues and Control at the Protein Level

Quantitative ELISA analysis revealed significant differences in protein concentrations between breast cancer subtypes and the control group (*p* < 0.05, Table 3). MAP3K1, PPM1D, LMTK3, and TGFB1 levels were significantly elevated across all tumor subtypes, with the highest concentrations consistently observed in TNBC (e.g., TGFB1: 544.92 ± 29.81 pg/mL; MAP3K1: 15.44 ± 0.56 ng/mL). In contrast, MAP2K4 and TP53 were significantly downregulated in tumors compared to controls, with the most pronounced reduction in TNBC subtypes.

### 2.5. Overall Survival

**Figure 4:**
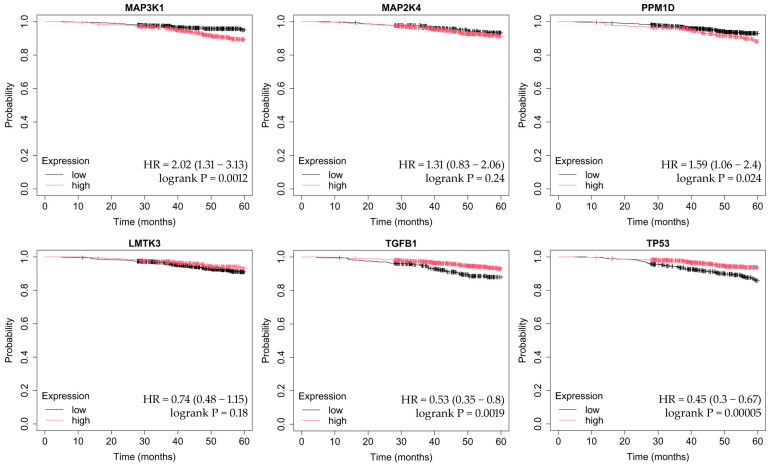
OS analysis in the luminal A breast cancer subtype. OS curves were generated using the Kaplan–Meier Plotter tool (http://kmplot.com; accessed on 27 July 2025). The analysis includes the following MAPK-associated genes: *MAP3K1*, *MAP2K4*, *PPM1D*, *LMTK3*, *TGFB1*, and *TP53*. High and low expression groups were stratified automatically based on the median cutoff. Hazard ratios (HRs) with 95% confidence intervals and log-rank *p*-values are shown according to the Kaplan–Meier Plotter output.

**Figure 5:**
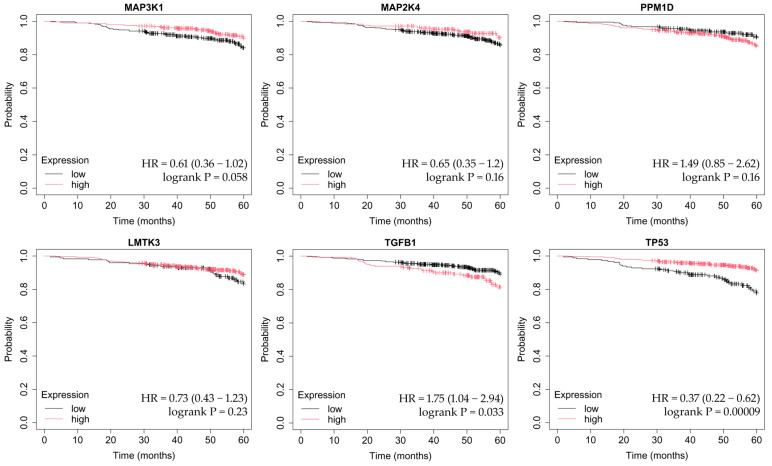
OS analysis in the HER2-negative luminal B breast cancer subtype. OS curves were generated using the Kaplan–Meier Plotter tool. (http://kmplot.com; accessed on 27 July 2025). The following MAPK-associated genes were evaluated: *MAP3K1*, *MAP2K4*, *PPM1D*, *LMTK3*, *TGFB1*, and *TP53*. Median expression was used as the cutoff for group stratification. HRs and log-rank *p*-values correspond to the Kaplan–Meier Plotter output.

**Figure 6:**
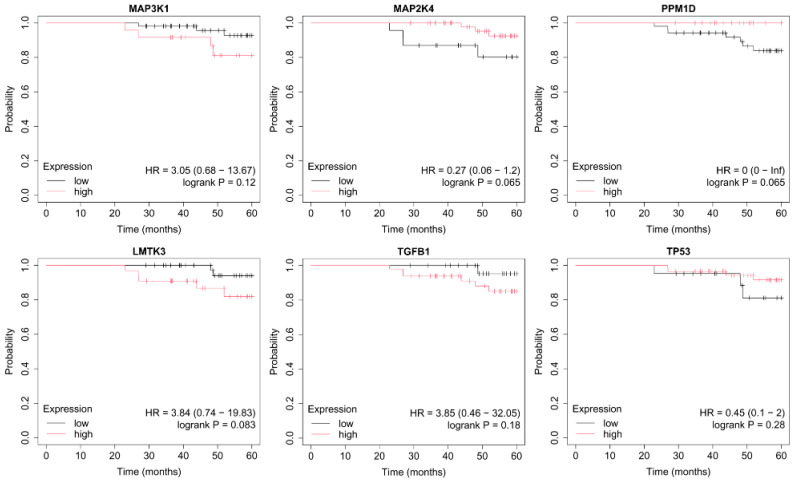
OS analysis in the HER2-positive luminal B subtype. OS curves were obtained using the Kaplan–Meier Plotter tool (http://kmplot.com; accessed on 27 July 2025). Genes included in the analysis were *MAP3K1*, *MAP2K4, PPM1D*, *LMTK3*, *TGFB1*, and *TP53.* Median-split stratification, hazard ratios, and log-rank *p*-values are reported according to the Kaplan–Meier Plotter tool.

**Figure 7:**
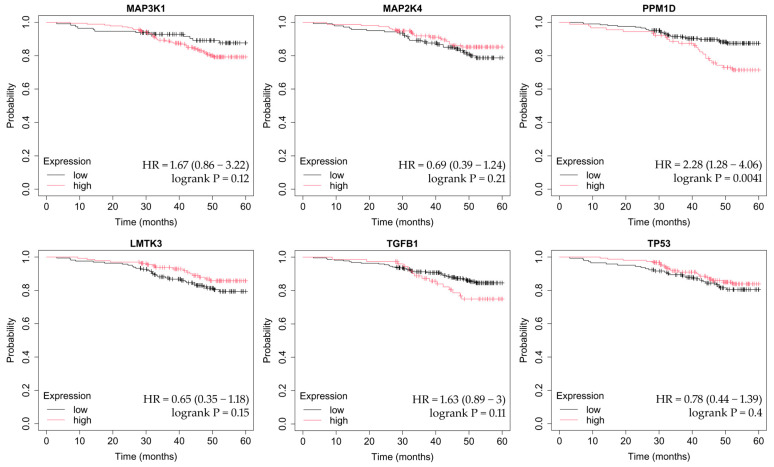
OS analysis in the non-luminal HER2-positive subtype. OS curves were calculated using the Kaplan–Meier Plotter tool. (http://kmplot.com; accessed on 27 July 2025). MAPK-associated genes included: *MAP3K1, MAP2K4*, *PPM1D*, *LMTK3*, *TGFB1*, and *TP53*. The plots show hazard ratios, confidence intervals, and log-rank *p*-values as provided by the Kaplan–Meier Plotter.

**Figure 8:**
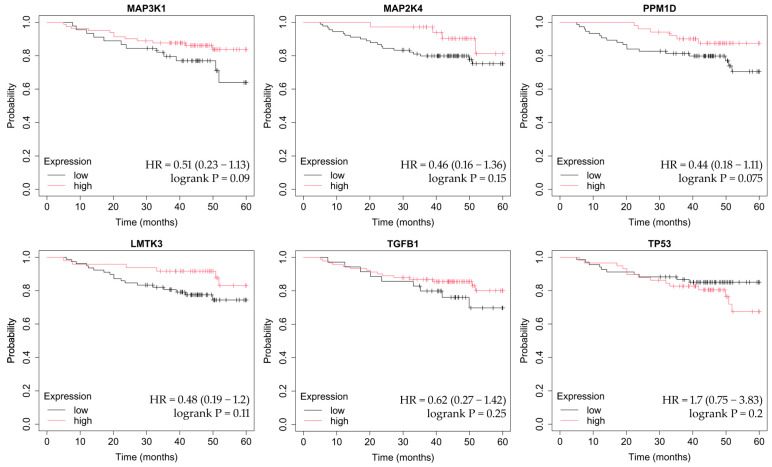
OS analysis in the TNBC subtype. OS curves were generated using the Kaplan–Meier Plotter tool (http://kmplot.com; accessed on 27 July 2025). The MAPK-associated genes included in the analysis were MAP3K1, MAP2K4, PPM1D, LMTK3, TGFB1, and TP53. Median expression was used to stratify high vs. low expression groups. HR values and log-rank statistics originate from the Kaplan–Meier Plotter.

OS was assessed using the Kaplan–Meier method, which estimates the probability of survival over time while accounting for censored observations. The Kaplan–Meier Plotter tool automatically stratifies patients into high- and low-expression groups (median cutoff) and calculates hazard ratios (HRs) with corresponding 95% confidence intervals, as well as log-rank *p*-values to determine statistical significance between survival curves. This approach allows visualization of how differential gene expression may influence long-term patient outcomes across breast cancer subtypes (Figure 4, Figure 5, Figure 6, Figure 7 and Figure 8).

In luminal A cancer, overexpression of *MAP3K1* and *PPM1D* and decreased expression of *TGFB1* and *TP53* are associated with poorer OS (Figure 4).

In HER2-negative luminal B cancer, overexpression of *TGFB1* and low expression of TP53 were associated with worse OS (Figure 5).

In HER2-positive luminal B cancer, changes in the expression of the analyzed genes did not affect OS (Figure 6).

In non-luminal HER2-positive cancer, *PPM1D* overexpression was associated with worse OS (Figure 7).

Changes in the expression of the studied genes did not significantly affect the deterioration of OS in TNBC (Figure 8).

RFS was also assessed for the studied genes and detailed data are provided in Table S2.

### 2.6. Functional Interaction Network and Enrichment Analysis of MAPK-Associated Genes

**Figure 9:**
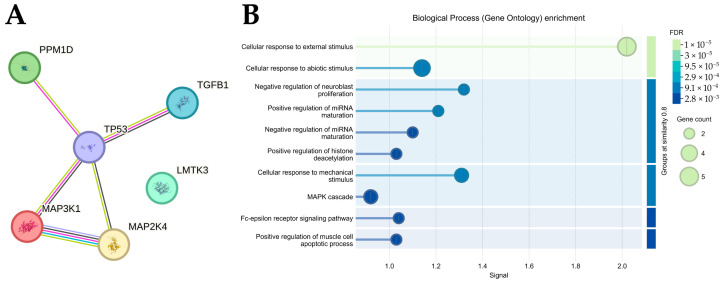
Functional Interaction Network and Enrichment Analysis of MAPK-Associated Genes. (**A**) STRING-based protein–protein interaction (PPI) network of six MAPK-related proteins (MAP3K1, MAP2K4, PPM1D, TP53, TGFB1, and LMTK3), illustrating 6 nodes and 5 edges, with TP53 as a central interaction hub. The observed connectivity exceeds random expectation (PPI enrichment *p* = 0.0344). (**B**) Enrichment analysis of overrepresented biological processes, highlighting signal transduction and MAPK-related pathways. Circle size represents gene count, color intensity. All pathway enrichment analyses were performed exclusively using differentially expressed genes identified in the present cohort.

The resulting network comprised 6 nodes and 5 edges, yielding an average node degree of 1.67 and an average local clustering coefficient of 0.694, indicating moderate interconnectivity. Notably, the observed number of edges (5) exceeded the expected number of edges (2) for a random set of proteins of similar size, reflecting a statistically significant enrichment of interactions (PPI enrichment *p*-value = 0.0344). This enrichment suggests that these proteins are not functionally independent and may be involved in coordinated biological processes. In the network, TP53 appeared as a central hub, directly interacting with MAP3K1, PPM1D, and TGFB1, consistent with its known role in coordinating stress and apoptotic signaling (Figure 9A). Complementary enrichment analysis (Figure 9B) identified significantly overrepresented biological processes (FDR-adjusted *p* < 0.05), with pathways related to signal transduction, MAPK cascade regulation, and cellular response to stress emerging as key categories. The gene count and false discovery rate (FDR) gradient reflect the robustness of these associations.

## 3. Discussion

Breast cancer is a heterogeneous disease characterized by molecular and clinical diversity, which demands an equally nuanced understanding of its regulatory networks [39,40,41]. In this study, we explored the molecular interplay between mitogen-activated protein kinase (MAPK)-associated genes and their regulatory miRNAs across five clinically relevant breast cancer subtypes. By integrating transcriptomic, proteomic, and miRNA expression data, we identified distinctive expression profiles for six MAPK-related genes—*MAP3K1*, *MAP2K4*, *PPM1D*, *LMTK3*, *TGFB1*, and *TP53*—and five miRNAs that may function as their upstream regulators. These findings illuminate the potential for subtype-specific MAPK-miRNA crosstalk to influence tumor behavior, therapeutic responsiveness, and prognosis [42]. To strengthen the external validity of our findings, we compared the expression profiles of the six MAPK-associated genes with data available in the UALCAN platform, which provides transcriptomic analyses derived from the TCGA-BRCA cohort. The overall expression trends observed in our study were strongly concordant with UALCAN-derived results: MAP3K1, MAP2K4, and TP53 were likewise reduced in tumor tissues—particularly in HER2-enriched and basal-like/TNBC subtypes—while PPM1D, LMTK3, and TGFB1 showed increased expression relative to normal controls. Small differences in the magnitude of expression shifts likely reflect methodological differences between datasets, including our use of microarray-based profiling versus RNA-Seq-derived TPM values in UALCAN, as well as differences in tumor stage distribution and ethnic composition between the cohorts. Nevertheless, the high directional consistency between our cohort and UALCAN supports the robustness of the subtype-specific MAPK dysregulation patterns identified in this study.

Our study extends the ongoing multi-pathway characterization of this well-defined breast cancer cohort. Whereas previous reports delineated dopaminergic, histaminergic, and SMAD/TGF-β [36,37,38] signaling perturbations, the present analysis focuses on MAPK-associated genes and their miRNA regulators, integrating transcriptional, translational, and survival data. This layered comparison reveals convergence between MAPK and TGF-β axes—particularly through MAP3K1, PPM1D, and TGFB1—that may underlie the aggressive phenotype of HER2+ and triple-negative subtypes.

Although our integrative analysis highlights potential crosstalk between MAPK-associated transcripts and regulatory miRNAs, these relationships are currently based on computational prediction and correlation analysis. No functional assays (e.g., miRNA mimic/inhibitor transfection, luciferase 3’UTR reporter assays, or AGO2-RIP/CLIP experiments) were performed in this study to confirm direct binding or regulatory effects. Future work should experimentally verify these predicted interactions to establish causal relationships and elucidate the precise mechanisms of MAPK–miRNA regulation in breast cancer subtypes.

Our investigation began with MAP3K1, a serine/threonine kinase that functions upstream in the MAPK signaling cascade. Its role is context-dependent, facilitating either pro-apoptotic or proliferative responses through JNK and ERK pathways [43,44]. In our cohort, MAP3K1 was consistently downregulated across all tumor subtypes, with the strongest suppression observed in TNBC. This attenuation may reflect a loss of stress-induced apoptosis in more aggressive cancers [45,46]. Interestingly, we also observed that *MAP3K1* overexpression was associated with worse OS in patients with luminal A cancer

Downregulation of *MAP3K1* coincided with a significant upregulation of miR-21-3p, a well-established oncomiR known to repress multiple tumor suppressor [47,48]. Concurrently, miR-23c, a predicted positive regulator of *MAP3K1*, was significantly decreased in TNBC. These opposing miRNA patterns suggest that *MAP3K1* expression is under coordinated post-transcriptional suppression, with implications for reduced apoptotic signaling in basal-like tumors [49].

Functionally downstream of MAP3K1 is MAP2K4, another dual-specificity kinase with tumor-suppressive properties via activation of JNK [19,50]. Similarly to MAP3K1, MAP2K4 was markedly downregulated at both transcript and protein levels, particularly in HER2-enriched and TNBC subtypes. The expression pattern of miR-27a-3p, which was significantly elevated in these subtypes, offers a compelling regulatory explanation. Known to promote proliferation and inhibit apoptosis, miR-27a-3p likely contributes to *MAP2K4* silencing, thereby weakening intrinsic stress responses [51]. The sequential downregulation of MAP3K1 and MAP2K4 across more aggressive subtypes emphasizes the dismantling of the canonical MAPK-mediated apoptotic arm [52,53,54].

In contrast, PPM1D (WIP1), a phosphatase that dampens the DNA damage response by deactivating p53 and other checkpoint proteins [55], was consistently overexpressed in all tumor subtypes, most notably in TNBC. This upregulation may support unchecked proliferation and resistance to genotoxic therapies [56,57,58]. Moreover, we observed that *PPM1D* overexpression was associated with worse OS in luminal A and non-luminal HER2-positive subtypes. miR-205-3p, a predicted repressor of *PPM1D*, demonstrated a subtype-dependent pattern—upregulated in luminal cancers but strongly suppressed in TNBC. This loss of inhibitory control in TNBC likely facilitates PPM1D overexpression and p53 inactivation. These molecular dynamics are particularly relevant given that both MAPK and p53 pathways intersect at the level of cellular stress regulation [59,60].

Complementing these findings, LMTK3—a kinase that stabilizes estrogen receptor alpha (ERα) and modulates chromatin accessibility—was markedly upregulated in all subtypes [61,62]. While its overexpression in luminal tumors is consistent with its role in estrogen signaling, its elevated levels in HER2-positive and TNBC subtypes suggest alternative, estrogen-independent roles, possibly via non-canonical MAPK Activation [63]. Although our study did not identify any regulatory miRNAs for LMTK3, its persistent upregulation across subtypes points to its broader relevance in promoting cell survival, endocrine resistance, and metastatic behavior [64].

The pleiotropic cytokine TGFB1 displayed strong overexpression at both mRNA and protein levels, especially in TNBC. TGFB1 is known to transition from a tumor suppressor in early tumorigenesis to a tumor promoter in advanced stages by inducing epithelial–mesenchymal transition (EMT), fibrosis, and immune evasion [65,66,67]. We observed that decreased *TGFB1* expression in luminal A cancer and its overexpression in HER2-negative luminal B cancer were associated with worse OS. While no miRNA regulator was confirmed in our panel, TGFB1’s established crosstalk with both MAPK and SMAD pathways [68,69] suggests it may act as a central hub for orchestrating stromal remodeling and metastasis, particularly in immune-cold or therapy-resistant tumors [64,65].

Finally, TP53, the master regulator of genomic integrity [70,71], was significantly downregulated across all tumor subtypes, most severely in TNBC [72,73,74,75]. Although TP53 emerged as the central node linking most MAPK-associated alterations in our cohort, this finding is not unexpected given its well-established role in genomic stability, stress signaling, and apoptosis. Indeed, the prominence of TP53 within the interaction network could be anticipated based on prior biological knowledge alone, independent of experimental discovery. The novelty of our study therefore does not lie in identifying TP53 itself, but rather in revealing the subtype-specific context in which TP53, MAPK components, and their putative miRNA regulators interact. In our cohort, the downregulation of miR-300, a predicted stabilizer of TP53 expression, occurred simultaneously with increased expression of *PPM1D*, an established p53 inhibitor, suggesting a dual mechanism of TP53 suppression: both transcriptional downregulation and loss of miRNA-mediated stabilization [76,77].

To further contextualize the identified MAPK-associated molecules, we generated a STRING interaction network and functional enrichment map. The PPI analysis showed that five of the six proteins—MAP3K1, MAP2K4, PPM1D, TGFB1, and TP53—form a connected interaction cluster, reflecting coordinated involvement in MAPK signaling, cellular stress responses, and apoptotic regulation. LMTK3 was included in the input list but did not meet the STRING confidence threshold for experimentally validated or high-probability interactions with the other proteins and therefore, appears as an orphan node and is not visualized in the network. This pattern indicates that while LMTK3 participates in MAPK-related biology through ER/MAPK crosstalk described in previous studies, its interactions are likely indirect or mediated through additional intermediates not captured within the STRING evidence model.

The enrichment of external-stimulus, stress-response, and apoptotic-response terms supports the notion that these signaling nodes integrate environmental and metabolic cues characteristic of aggressive breast-cancer subtypes. Placing our findings within canonical cascades clarifies their functional relevance: MAP3K1 (MEKK1) activates MAP2K4 (MKK4) and MAP2K7 (MKK7) to stimulate the JNK pathway; MAP2K1/2 (MEK1/2) drive ERK activation; and TAK1 (MAP3K7) together with MAP2K3/6 (MKK3/6) modulate p38 signaling. PPM1D (WIP1) dephosphorylates p38/JNK and regulates TP53, establishing a stress-response feedback loop, whereas TGFB1 engages both canonical SMAD-dependent and non-canonical MAPK branches. Together, these relationships emphasize the centrality of MAPK stress circuitry in shaping the molecular phenotype of the examined breast-cancer subtypes, while also highlighting the unique positioning of LMTK3 as a MAPK-associated but not directly interconnected component.

Collectively, our subtype-specific expression/protein patterns suggest ERK-leaning circuitry in luminal disease and greater JNK/p38 stress-axis engagement in aggressive subtypes, with PPM1D–TP53 feedback as a shared vulnerability. [78]. Integration of the miRNA expression data reinforces this model by adding a post-transcriptional layer of complexity that helps explain the observed protein-level alterations [79].

This study has several limitations that should be acknowledged. First, although the cohort was prospectively collected, it is the same patient population used in our previous pathway-focused analyses (histaminergic, dopaminergic, and SMAD/TGF-β), which may limit external generalizability despite the inclusion of entirely new MAPK-specific transcriptomic, miRNA, proteomic, and survival analyses in the present work. Second, the regulatory relationships between dysregulated miRNAs and MAPK-associated genes were inferred from bioinformatic prediction tools and inverse expression patterns; however, no mechanistic experiments—such as miRNA gain- or loss-of-function assays, luciferase 3′UTR reporter validation, AGO2-RIP, or CLIP-seq—were performed to confirm direct binding or functional regulation. These interactions should therefore be considered hypothesis-generating. Third, while STRING-based protein–protein interaction and enrichment analyses provided meaningful insights into pathway connectivity, these tools cannot fully capture dynamic, context-dependent signaling interactions, post-translational modifications, or epigenetic mechanisms that shape MAPK pathway activity in breast cancer. Fourth, although the overall sample size was adequate, stratification into five molecular subtypes—particularly HER2-positive and TNBC—reduced the statistical power of survival analyses and may have obscured weaker prognostic effects. Finally, external validation relied on publicly available transcriptomic data (TCGA-BRCA/UALCAN), which differ in demographic composition, sequencing methodology, and tumor-stage distribution from our cohort. Larger multicenter datasets, together with functional studies, will be necessary to fully validate the subtype-specific MAPK–miRNA regulatory axes identified here.

## 4. Materials and Methods

The present study was conducted using the same prospectively collected cohort of 405 Polish women with histologically confirmed breast cancer that has previously served as the basis for three molecular pathways –focused analyses: the histaminergic system, the dopaminergic system, and SMAD/TGF-β signaling [36,37,38]. All demographic and clinicopathological data, as well as inclusion/exclusion criteria, were identical to those in the previous studies and are reproduced here for consistency. However, all analyses of MAPK-associated mRNAs, their regulatory miRNAs, protein quantification, network interactions, and survival correlations are first reported in this manuscript. A summary comparing reused and novel endpoints is presented in Supplementary Table S1.

### 4.1. Participants

The study enrolled 405 patients with breast cancer, categorized into five molecular subtypes: 130 with luminal A, 100 with HER2-negative luminal B, 96 with HER2-positive luminal B, 36 with non-luminal HER2-positive, and 43 with triple-negative breast cancer (TNBC). Margin samples of healthy tissue collected during surgery served as controls. All tumor and control tissues were classified as neoplastic or non-neoplastic based on pathological evaluation. Each patient was staged as T1N0M0.

Across subtypes, tumor grades varied: in luminal A, 18% were G1, 37% G2, and 45% G3; in HER2-negative luminal B, 31% were G1, 57% G2, and 12% G3; in HER2-positive luminal B, 24% were G1, 59% G2, and 17% G3. For non-luminal HER2-positive tumors, 25% were G1, 33% G2, and 42% G3, while TNBC cases comprised 32% G1, 49% G2, and 19% G3. Age distribution showed that in luminal A, 33% of patients were under 50 years, compared to 32% in HER2-negative luminal B, 20% in HER2-positive luminal B, 25% in non-luminal HER2-positive, and 23% in TNBC. The remaining majority in each group were over 50 years old. Average BMI values were as follows: 30.78 ± 2.76 kg/m^2^ in luminal A, 30.18 ± 4.56 kg/m^2^ in HER2-negative luminal B, 32.09 ± 6.19 kg/m^2^ in HER2-positive luminal B, 33.18 ± 5.67 kg/m^2^ in non-luminal HER2-positive, and 34.67 ± 2.98 kg/m^2^ in TNBC.

The study adhered to the principles of the 2013 Declaration of Helsinki and received ethical approval from the Bioethical Committee of the Regional Medical Chamber in Krakow (81/KBL/OIL/2023) on 10 March 2023. Informed consent was obtained from all participants.

### 4.2. Isolation of Total Ribonucleic Acid (RNA) from Tissues

Total RNA was extracted using TRIzol reagent (Invitrogen, Carlsbad, CA, USA, cat. no. 15596026) and purified with the RNeasy Mini Kit (QIAGEN, Hilden, Germany, cat. no. 74104, cat. no. 74104) and DNase I (Fermentas International Inc., Burlington, ON, Canada; cat. no. 18047019). RNA quality and quantity were assessed by 1% agarose gel electrophoresis and spectrophotometric absorbance measurement.

### 4.3. mRNA Microarray Analysis

Transcriptome profiling was performed using HG-U133A 2.0 microarrays (Affymetrix, Santa Clara, CA, USA) in combination with the GeneChip™ 3′IVT PLUS Kit (Thermo Fisher Scientific, Inc., Waltham, MA, USA, cat. no. 902416). A gene list was generated based on the MAPK signaling pathway (hsa04010) from the Kyoto Encyclopedia of Genes and Genomes (KEGG) database (http://pathcards.genecards.org/ accessed on 1 March 2025). The same microarray dataset previously used to explore SMAD, dopamine, and histamine signaling was re-queried here using KEGG pathway enrichment to isolate 312 MAPK-related genes. Differentially expressed genes were validated by Reverse Transcription Quantitative Polymerase Chain Reaction (RT-qPCR) and Enzyme-Linked Immunosorbent Assay (ELISA) and integrated with miRNA expression data to delineate subtype-specific MAPK regulatory modules (Table S1 summarizes previous versus new analyses).

### 4.4. RT-qPCR Validation of Microarray Results

Six genes with significant differential expression across subtypes were selected for validation. RT-qPCR was performed using the SensiFast SYBR No-ROX One-Step Kit (Bioline, London, UK), with β-actin (*ACTB*) as the reference gene. Relative expression levels were calculated using the 2^−ΔΔCt^ method. The primers’ sequence is shown in Table 4. RT-qPCR was performed for all study and control samples.

### 4.5. External Validation Using UALCAN Platform

External transcriptomic data were obtained from the UALCAN portal (https://ualcan.path.uab.edu; first accessed on 26 November 2025) to validate RNA expression patterns identified in our cohort [80]. UALCAN provides normalized RNA-seq expression profiles derived from the TCGA Breast Invasive Carcinoma dataset (TCGA-BRCA). For each MAPK-associated gene analyzed in this study (*MAP3K1*, *MAP2K4*, *PPM1D*, *LMTK3*, *TGFB1*, *TP53*), two levels of comparison were performed.

First, expression levels in primary breast cancer samples were compared with normal breast tissue using UALCAN’s standard tumor–normal module.

Second, subtype-resolved comparisons were extracted using UALCAN’s molecular classification panels. These included normal breast tissue, luminal tumors, HER2-positive tumors, and the full spectrum of triple-negative breast cancer (TNBC) subclasses defined in TCGA-BRCA:

TNBC-BL1 (Basal-like 1), TNBC-BL2 (Basal-like 2), TNBC-IM (Immunomodulatory), TNBC-M (Mesenchymal), TNBC-MSL (Mesenchymal Stem-Like), TNBC-LAR (Luminal Androgen Receptor), and TNBC-UNS (Unspecified).

For each queried gene, expression values were retrieved directly from UALCAN’s gene-specific query module and recorded as normalized TPM or read-count-based values as provided by the platform. The external TCGA-derived data were used exclusively for descriptive comparison of distribution patterns with the microarray results from our cohort, without additional statistical reprocessing.

### 4.6. Protein Quantification by ELISA

Protein expression levels were quantified using ELISA (Abbexa, Cambridge, UK) with commercially available kits from MyBioSource, Inc. (San Diego, CA, USA). The following kits were used: MAP3K1 (Mitogen-Activated Protein Kinase Kinase Kinase 1; catalog no. MBS9303590), MAP2K4 (Dual Specificity Mitogen-Activated Protein Kinase Kinase 4; catalog no. MBS1606121), PPM1D (Protein Phosphatase, Mg^2+^/Mn^2+^ Dependent 1D; catalog no. MBS2707714), TP53 (Tumor Protein P53; catalog no. MBS041908), TGFB1 (Transforming Growth Factor Beta 1; catalog no. MBS2023574), and LMTK3 (Lemur Tyrosine Kinase 3; catalog no. MBS2705460). All assays were conducted according to the manufacturers’ instructions.

### 4.7. miRNA Profiling and Target Prediction

Potential miRNA regulators of the differentially expressed MAPK-associated genes were identified using the miRDB database (accessed on 5 May 2025), applying a high-confidence prediction score cutoff of ≥80. For each MAPK-related gene, all predicted miRNAs meeting this criterion were retrieved and subsequently compared with the miRNA expression profiles obtained from our patient cohort. Only miRNAs that were both predicted with high confidence and experimentally detected as significantly dysregulated (*p* < 0.05; |log2FC| > 2) were retained. To identify the most plausible regulatory relationships, we focused on predicted miRNA–mRNA pairs that showed opposite directions of expression, consistent with canonical miRNA-mediated repression. This approach provides in silico, expression-based indications of potential regulatory interactions; however, it does not constitute direct experimental confirmation of miRNA–mRNA binding, and the identified pairs should therefore be regarded as hypothesis-generating [81].

### 4.8. Statistical Analysis

Microarray data were analyzed using the Transcriptome Analysis Console (Thermo Fisher Scientific, Waltham, MA, USA). Significance was determined by one-way ANOVA followed by Tukey’s post hoc test (*p* < 0.05; |fold change| > 2). RT-qPCR and ELISA data were analyzed in Statistica 13.3 (StatSoft, Krakow, Poland). The Shapiro–Wilk test was used to assess normality; due to non-parametric distributions, Kruskal–Wallis and Dunn’s post hoc tests were applied.

To ensure adequate statistical power for transcriptomic and molecular subgroup analyses, an a priori sample size calculation was performed using G*Power 3.1 software [82]. Based on national epidemiological data from Poland reporting approximately 19,620 new breast cancer cases in 2019 [83], a minimum sample size of 377 patients was estimated to achieve a 95% confidence level and a 5% margin of error. To meet this criterion and ensure sufficient subgroup representation, the study enrolled 405 female patients with histopathologically confirmed breast cancer [84]. Overall survival (OS) analyses for each gene and breast cancer subtype were performed using the Kaplan–Meier Plotter tool (http://kmplot.com; accessed on 27 July 2025), with the follow-up period restricted to 60 months. Hazard ratios (HRs), 95% confidence intervals (95% CI), and log-rank *p*-values generated by KMplot are shown directly on each survival curve and were used as the statistical output for the survival assessment. Recurrence-Free Survival (RFS) analyses were also performed using the Kaplan–Meier Plotter tool (http://kmplot.com; accessed on 28 November 2025) with a 60-month follow-up threshold. HRs, CI and *p*-values are presented in Table S2 [85,86].

Protein–protein interactions (PPI) were comprehensively analyzed using the STRING database (version 11.0; accessed on 27 July 2025). STRING evaluates functional associations by calculating the enrichment strength, expressed as Log_10_(observed/expected)—a metric that compares the number of proteins associated with a given annotation in the input network to the number expected in a random network of similar size. A higher value indicates stronger enrichment. To assess statistical significance, false discovery rates (FDRs) were calculated using the Benjamini–Hochberg correction for multiple comparisons, providing adjusted *p*-values for each functional category [87].

## 5. Conclusions

In summary, our findings reveal a coherent but subtype-specific dysregulation of MAPK-associated gene expression in breast cancer, with miRNAs playing a pivotal regulatory role. The most pronounced transcriptional and post-transcriptional alterations were observed in TNBC, highlighting potential pathways for therapeutic targeting. Understanding these molecular relationships may inform the development of subtype-tailored therapies aimed at reactivating tumor-suppressive signaling or disrupting oncogenic feedback loops.

## Figures and Tables

**Table 1:** Differential Expression of Selected MAPK-Related Genes Across Breast Cancer Subtypes Compared to Control Tissue.

ID	mRNA	Log_2_ Fold Change				
		LumA vs. C	LumB HER2− vs. C	LumB HER2+ vs. C	HER2+ vs. C	TNBC vs. C
214786_at	*MAP3K1*	−2.87	−3.14	−3.65	−3.76	−4.32
225927_at		−2.19	−3.25	−3.71	−3.19	−4.61
203265_s_at	*MAP2K4*	−3.43	−4.51	−4.71	−4.65	3.89
203266_s_at		−3.18	−4.41	−4.76	−4.81	3.81
204566_at	*PPM1D*	2.71	2.98	3.45	4.18	4.87
230330_at		2.19	3.12	3.19	4.09	4.56
1557103_a_at	*LMTK3*	4.56	4.71	4.98	4.88	2.19
203084_at	*TGFB1*	4.80	4.43	4.07	2.97	5.18
203085_s_at		4.00	4.33	3.31	2.17	4.10
201746_at	*TP53*	−3.01	−3.87	−3.71	−3.99	−5.65
211300_s_at		−3.16	−3.52	−3.32	−4.09	−5.71

Log_2_Fold-change values are shown; positive values indicate upregulation in tumor tissue relative to control, negative values indicate downregulation. ID, number of the probe; LumA, luminal A; LumB, luminal B; HER2, human epidermal growth factor receptor 2; TNBC, triple-negative breast cancer; C, control; *MAP3K1* (Mitogen-Activated Protein Kinase Kinase Kinase 1); *MAP2K4* (Mitogen-Activated Protein Kinase Kinase 4); *PPM1D* (Protein Phosphatase, Mg2+/Mn2+ Dependent 1D (also known as *WIP1*); *LMTK3* (Lemur Tyrosine Kinase 3); *TGFB1* (Transforming Growth Factor Beta 1); *TP53* (Tumor Protein p53).

**Table 2:** Significantly Dysregulated miRNAs Predicted to Target MAPK Pathway Genes (*p* < 0.05; |Fold Change| > 2).

mRNA	miRNA	Target Score	Log2 Fold Change				
			LumA vs. C	LumB HER2− vs. C	LumB HER2+ vs. C	HER2+ vs. C	TNBC
*MAP3K1*	hsa-miR-21-3p	94	2.11 ± 0.41 *	2.65 ± 0.18 *	2.45 ± 0.32 *	2.91 ± 0.12 *	3.19 ± 0.65 *
	hsa-miR-23c	97	−3.12 ± 0.56 *	−3.54 ± 0.71 *	−3.19 ± 0.61 *	−2.87 ± 0.87 *	−3.88 ± 0.71 *
*MAP2K4*	hsa-miR-27a-3p	92	3.12 ± 0.71 *	3.19 ± 0.87 *	2.99 ± 0.81 *	4.51 ± 0.19 *	4.81 ± 0.91 *
*PPM1D*	hsa-miR-205-3p	90	2.91 ± 0.76 *	2.01 ± 0.22 *	−3.41 ± 0.91 *	−3.17 ± 0.81 *	−5.91 ± 1.07 *
*TP53*	hsa-miR-300	89	−3.12 ± 0.17 *	−3.87 ± 0.65 *	−4.16 ± 0.91 *	−4.12 ± 0.78 *	−5.61 ± 1.01 *

Log_2_Fold-change values are shown. Data are presented as mean ± SD; positive values indicate upregulation in tumor tissue relative to control, negative values indicate downregulation * *p* < 0.05 vs. control.

**Table 3:** Concentration of selected proteins in breast cancer subtypes and control group (*p* < 0.05).

Protein	Control	LumA	LumB HER2−	LumB HER2+	HER2+	TNBC
MAPK3K1 [ng/mL]	3.45 ± 0.31	5.87 ± 0.43 *	7.67 ± 0.81 *	10.23 ± 0.81 *	11.23 ± 1.92 *	15.44 ± 0.56 *
MAP2K4 [ng/mL]	581.92 ± 17.71	321.94 ± 7.65 *	316.71 ± 10.41 *	272.12 ± 11.61 *	200.12 ± 12.23 *	225.12 ± 18.81 *
PPM1D [ng/mL]	1.12 ± 0.18	3.91 ± 0.92 *	4.18 ± 0.54 *	5.06 ± 0.61 *	5.87 ± 0.91 *	7.13 ± 0.42 *
LMTK3 [ng/mL]	0.47 ± 0.03	0.91 ± 0.23 *	1.23 ± 0.26 *	4.32 ± 0.89 *	4.96 ± 0.54 *	6.79 ± 0.19 *
TGFB1 [pg/mL]	79.81 ± 2.13	169.18 ± 5.65 *	198.98 ± 12.56 *	331.98 ± 32.19 *	391.76 ± 40.16 *	544.92 ± 29.81 *
TP53 [pg/mL]	1341.01 ± 18.19	561.91 ± 10.91 *	544.91 ± 9.61 *	577.19 ± 9.1 8*	918.81 ± 54.12 *	1287.19 ± 31.41 *

Data are presented as mean ± SD. * *p* < 0.05 vs. control.

**Table 4:** Forward and Reverse Primer Sequences for Gene Expression Analysis.

mRNA	Nucleotide Sequence	
*MAP3K1*	Forward	5′-CCACAGAGAACAGTTCCCCT-3′
	Reverse	5′-CCATTGGCTTTGGTTGCTCT-3′
*MAP2K4*	Forward	5′-TCGGGCTTGAGTGAGAAGAG-3′
	Reverse	5′-GAGCACATCGATCCCCAAAC-3′
*PPM1D*	Forward	5′-TGGGTGAGCATGGACAATCT-3′
	Reverse	5′-GGTGGTGTAGAACATGGGGA-3′
*LMTK3*	Forward	5′-CCTTCGTGGTTCAAGTGAGC-3′
	Reverse	5′-CCCGGGACTTTCTCTCTGTT-3′
*TGFB1*	Forward	5′-TGAACCGGCCTTTCCTGCTTCTCATG-3′
	Reverse	5′-CGGAAGTCAATGTACAGCTGCCGC-3′
*TP53*	Forward	5′-TGGCCATCTACAAGCAGTCA-3′
	Reverse	5′-CGGTACAGTCAGAGCCAACCT-3′
	Reverse	5′-CGGAAGTCAATGTACAGCTGCCGC-3′
*ACTB*	Forward	5′-TCACCCACACTGTGCC CATCTACGA-3′
	Reverse	5′-CAGCGGAACCGCTCATTGCCAATGG-3′

## Data Availability

The data used to support findings of this study are included in this article. The data will not be shared due to third-party rights and commercial confidentiality.

## Supplementary Materials

The following supporting information can be downloaded at: https://www.mdpi.com/article/10.3390/ijms262411831/s1.

## Abbreviations

The following abbreviations are used in this manuscript:

Abbreviation	Full Term
ACTB	Beta-Actin
ANOVA	Analysis of Variance
BC	Breast Cancer
BMI	Body Mass Index
C	Control
DNA	Deoxyribonucleic Acid
ELISA	Enzyme-Linked Immunosorbent Assay
EMT	Epithelial–Mesenchymal Transition
ER	Estrogen Receptor
ERK	Extracellular Signal-Regulated Kinase
FDR	False Discovery Rate
G*Power	General Power Analysis Program
HER2	Human Epidermal Growth Factor Receptor 2
IHC	Immunohistochemistry
JNK	c-Jun N-terminal Kinase
KEGG	Kyoto Encyclopedia of Genes and Genomes
LMTK3	Lemur Tyrosine Kinase 3
Log_2_FC	Log_2_ Fold Change
LumA	Luminal A Breast Cancer Subtype
LumB HER2−	HER2-Negative Luminal B Breast Cancer Subtype
LumB HER2+	HER2-Positive Luminal B Breast Cancer Subtype
MAP2K4	Mitogen-Activated Protein Kinase Kinase 4
MAP3K1	Mitogen-Activated Protein Kinase Kinase Kinase 1
MAPK	Mitogen-Activated Protein Kinase
miRNA	microRNA
mRNA	Messenger Ribonucleic Acid
OS	Overall Survival
PCR	Polymerase Chain Reaction
PPI	Protein–Protein Interaction
PPM1D	Protein Phosphatase, Mg^2+^/Mn^2+^ Dependent 1D (WIP1)
PR	Progesterone Receptor
RNA	Ribonucleic Acid
RT-qPCR	Reverse Transcription Quantitative Polymerase Chain Reaction
SD	Standard Deviation
SMAD	Mothers Against Decapentaplegic Homolog
STRING	Search Tool for the Retrieval of Interacting Genes/Proteins
TGFB1	Transforming Growth Factor Beta 1
TNBC	Triple-Negative Breast Cancer
TP53	Tumor Protein p53
TGF-β	Transforming Growth Factor Beta
WIP1	Wild-Type p53-Induced Phosphatase 1

## References

[1] Sung H., Ferlay J., Siegel R.L., Laversanne M., Soerjomataram I., Jemal A., Bray F. (2021). Global Cancer Statistics 2020: GLOBOCAN Estimates of Incidence and Mortality Worldwide for 36 Cancers in 185 Countries. CA Cancer J. Clin..

[2] Liu Y.-B., Gao X.-T., Huang L.-Y., Liu X.-L. (2024). Clinicopathological Characteristics and Prognostic Factors in Invasive Micropapillary Carcinoma of the Breast. Arch. Med. Sci..

[3] Testa U., Castelli G., Pelosi E. (2020). Breast Cancer: A Molecularly Heterogenous Disease Needing Subtype-Specific Treatments. Med. Sci..

[4] Rakha E.A., Tse G.M., Quinn C.M. (2023). An Update on the Pathological Classification of Breast Cancer. Histopathology.

[5] Tsang J.Y., Gary M.T. (2020). Molecular Classification of Breast Cancer. Adv. Anat. Pathol..

[6] Lashen A.G., Toss M.S., Mongan N.P., Green A.R., Rakha E.A. (2023). The Clinical Value of Progesterone Receptor Expression in Luminal Breast Cancer: A Study of a Large Cohort with Long-Term Follow-Up. Cancer.

[7] Wang J., Li B., Luo M., Huang J., Zhang K., Zheng S., Zhang S., Zhou J. (2024). Progression from Ductal Carcinoma in Situ to Invasive Breast Cancer: Molecular Features and Clinical Significance. Sig. Transduct. Target. Ther..

[8] Maqsood Q., Khan M.U., Fatima T., Khalid S., Malik Z.I. (2025). Recent Insightsinto Breast Cancer: Molecular Pathways, Epigenetic Regulation, and Emerging Targeted Therapies. Breast Cancer.

[9] Lavasani M.A., Moinfar F. (2012). Molecular Classification of Breast Carcinomas with Particular Emphasis on “Basal-like” Carcinoma: A Critical Review. J. Biophotonics.

[10] Denkert C., Rachakonda S., Karn T., Weber K., Martin M., Marmé F., Untch M., Bonnefoi H., Kim S.-B., Seiler S. (2025). Dynamics of Molecular Heterogeneity in High-Risk Luminal Breast Cancer—From Intrinsic to Adaptive Subtyping. Cancer Cell.

[11] Caldarella A., Crocetti E., Bianchi S., Vezzosi V., Urso C., Biancalani M., Zappa M. (2011). Female Breast Cancer Status According to ER, PR and HER2 Expression: A Population Based Analysis. Pathol. Oncol. Res..

[12] Corti C., De Angelis C., Bianchini G., Malorni L., Giuliano M., Hamilton E., Jeselsohn R., Jhaveri K., Curigliano G., Criscitiello C. (2023). Novel Endocrine Therapies: What Is next in Estrogen Receptor Positive, HER2 Negative Breast Cancer?. Cancer Treat. Rev..

[13] Jin X., Zhou Y.-F., Ma D., Zhao S., Lin C.-J., Xiao Y., Fu T., Liu C.-L., Chen Y.-Y., Xiao W.-X. (2023). Molecular Classification of Hormone Receptor-Positive HER2-Negative Breast Cancer. Nat. Genet..

[14] Kumar N., Gann P.H., McGregor S.M., Sethi A. (2023). Quantification of Subtype Purity in Luminal A Breast Cancer Predicts Clinical Characteristics and Survival. Breast Cancer Res. Treat..

[15] Lafcı O., Celepli P., Öztekin P.S., Koşar P.N. (2023). DCE-MRI Radiomics Analysis in Differentiating Luminal a and Luminal B Breast Cancer Molecular Subtypes. Acad. Radiol..

[16] Yang Z., Liu Y., Huang Y., Chen Z., Zhang H., Yu Y., Wang X., Cao X. (2023). The Regrouping of Luminal B (HER2 Negative), a Better Discriminator of Outcome and Recurrence Score. Cancer Med..

[17] Falato C., Schettini F., Pascual T., Brasó-Maristany F., Prat A. (2023). Clinical Implications of the Intrinsic Molecular Subtypes in Hormone Receptor-Positive and HER2-Negative Metastatic Breast Cancer. Cancer Treat. Rev..

[18] Thomas A., Reis-Filho J.S., Geyer C.E., Wen H.Y. (2023). Rare Subtypes of Triple Negative Breast Cancer: Current Understanding and Future Directions. NPJ Breast Cancer.

[19] Liu S., Huang J., Zhang Y., Liu Y., Zuo S., Li R. (2019). MAP2K4 Interacts with Vimentin to Activate the PI3K/AKT Pathway and Promotes Breast Cancer Pathogenesis. Aging.

[20] Asl E.R., Amini M., Najafi S., Mansoori B., Mokhtarzadeh A., Mohammadi A., Lotfinejad P., Bagheri M., Shirjang S., Lotfi Z. (2021). Interplay between MAPK/ERK Signaling Pathway and MicroRNAs: A Crucial Mechanism Regulating Cancer Cell Metabolism and Tumor Progression. Life Sci..

[21] Stefani C., Miricescu D., Stanescu-Spinu I.-I., Nica R.I., Greabu M., Totan A.R., Jinga M. (2021). Growth Factors, PI3K/AKT/mTOR and MAPK Signaling Pathways in Colorectal Cancer Pathogenesis: Where Are We Now?. Int. J. Mol. Sci..

[22] Braicu C., Buse M., Busuioc C., Drula R., Gulei D., Raduly L., Rusu A., Irimie A., Atanasov A.G., Slaby O. (2019). A Comprehensive Review on MAPK: A Promising Therapeutic Target in Cancer. Cancers.

[23] Gugliandolo A., Silvestro S., Sindona C., Bramanti P., Mazzon E. (2021). MiRNA: Involvement of the MAPK Pathway in Ischemic Stroke. A Promising Therapeutic Target. Medicina.

[24] Correia de Sousa M., Gjorgjieva M., Dolicka D., Sobolewski C., Foti M. (2019). Deciphering miRNAs’ Action through miRNA Editing. Int. J. Mol. Sci..

[25] Safa A., Abak A., Shoorei H., Taheri M., Ghafouri-Fard S. (2020). MicroRNAs as Regulators of ERK/MAPK Pathway: A Comprehensive Review. Biomed. Pharmacother..

[26] Otmani K., Lewalle P. (2021). Tumor Suppressor miRNA in Cancer Cells and the Tumor Microenvironment: Mechanism of Deregulation and Clinical Implications. Front. Oncol..

[27] Reddy K.B. (2015). MicroRNA (miRNA) in Cancer. Cancer Cell Int..

[28] Ye J., Xu M., Tian X., Cai S., Zeng S. (2019). Research Advances in the Detection of miRNA. J. Pharm. Anal..

[29] Lao D.T., Quang M.T., Le T.A.H. (2021). The Role of Hsa-miR-21 and Its Target Genes Involved in Nasopharyngeal Carcinoma. Asian Pac. J. Cancer Prev..

[30] Plawgo K., Raczynska K.D. (2022). Context-Dependent Regulation of Gene Expression by Non-Canonical Small RNAs. Noncoding RNA.

[31] Namdeo P.K., Mishra S., Das A., Tiwari R.R., Subbiah R. (2025). The Interplay between microRNAs and Oxidative Stress and Its Implications in Respiratory Diseases. Free Radic. Res..

[32] Chai D., Du H., Shi Y. (2025). The Progress of miR-205 Regulating Apoptosis in Cancer. Front. Oncol..

[33] Xiao Y., Humphries B., Yang C., Wang Z. (2019). MiR-205 Dysregulations in Breast Cancer: The Complexity and Opportunities. Noncoding RNA.

[34] Shi C., Zhang Z. (2017). Screening of Potentially Crucial Genes and Regulatory Factors Involved in Epithelial Ovarian Cancer Using Microarray Analysis. Oncol. Lett..

[35] Maginnis M.S. (2023). β-Arrestins and G Protein-Coupled Receptor Kinases in Viral Entry: A Graphical Review. Cell. Signal..

[36] Sirek T., Sirek A., Borawski P., Zmarzły N., Sułkowska J., Król-Jatręga K., Opławski M., Boroń D., Chalcarz M., Ossowski P. (2024). miRNAs in Signal Transduction of SMAD Proteins in Breast Cancer. Int. J. Mol. Sci..

[37] Sirek T., Sirek A., Opławski M., Boroń D., Chalcarz M., Ossowski P., Dziobek K., Zmarzły N., Strojny D., Grabarek B.O. (2024). Expression Profile of Messenger and Micro RNAs Related to the Histaminergic System in Patients with Five Subtypes of Breast Cancer. Front. Oncol..

[38] Sirek T., Sirek A., Borawski P., Ryguła I., Król-Jatręga K., Opławski M., Boroń D., Chalcarz M., Ossowski P., Dziobek K. (2024). Expression Profiles of Dopamine-Related Genes and miRNAs Regulating Their Expression in Breast Cancer. Int. J. Mol. Sci..

[39] Salemme V., Centonze G., Avalle L., Natalini D., Piccolantonio A., Arina P., Morellato A., Ala U., Taverna D., Turco E. (2023). The Role of Tumor Microenvironment in Drug Resistance: Emerging Technologies to Unravel Breast Cancer Heterogeneity. Front. Oncol..

[40] Andrade de Oliveira K., Sengupta S., Yadav A.K., Clarke R. (2023). The Complex Nature of Heterogeneity and Its Roles in Breast Cancer Biology and Therapeutic Responsiveness. Front. Endocrinol..

[41] Eiro N., Gonzalez L.O., Fraile M., Cid S., Schneider J., Vizoso F.J. (2019). Breast Cancer Tumor Stroma: Cellular Components, Phenotypic Heterogeneity, Intercellular Communication, Prognostic Implications and Therapeutic Opportunities. Cancers.

[42] Ullah R., Yin Q., Snell A.H., Wan L. (2022). RAF-MEK-ERK Pathway in Cancer Evolution and Treatment. Semin. Cancer Biol..

[43] Yue J., López J.M. (2020). Understanding MAPK Signaling Pathways in Apoptosis. Int. J. Mol. Sci..

[44] Cai Y.-W., Liu C.-C., Zhang Y.-W., Liu Y.-M., Chen L., Xiong X., Shao Z.-M., Yu K.-D. (2024). MAP3K1 Mutations Confer Tumor Immune Heterogeneity in Hormone Receptor-Positive HER2-Negative Breast Cancer. J. Clin. Investig..

[45] Hong Y., He J., Deng D., Liu Q., Zu X., Shen Y. (2025). Targeting Kinases That Regulate Programmed Cell Death: A New Therapeutic Strategy for Breast Cancer. J. Transl. Med..

[46] Iuliano L., Dalla E., Picco R., Mallavarapu S., Minisini M., Malavasi E., Brancolini C. (2022). Proteotoxic Stress-Induced Apoptosis in Cancer Cells: Understanding the Susceptibility and Enhancing the Potency. Cell Death Discov..

[47] Otmani K., Rouas R., Lewalle P. (2022). OncomiRs as Noncoding RNAs Having Functions in Cancer: Their Role in Immune Suppression and Clinical Implications. Front. Immunol..

[48] Nguyen H.T., Kacimi S.E.O., Nguyen T.L., Suman K.H., Lemus-Martin R., Saleem H., Do D.N. (2021). MiR-21 in the Cancers of the Digestive System and Its Potential Role as a Diagnostic, Predictive, and Therapeutic Biomarker. Biology.

[49] van den Ende N.S., Nguyen A.H., Jager A., Kok M., Debets R., van Deurzen C.H.M. (2023). Triple-Negative Breast Cancer and Predictive Markers of Response to Neoadjuvant Chemotherapy: A Systematic Review. Int. J. Mol. Sci..

[50] Liu X.-D., Zhang Z.-W., Wu H.-W., Liang Z.-Y. (2021). A New Prognosis Prediction Model Combining TNM Stage with MAP2K4 and JNK in Postoperative Pancreatic Cancer Patients. Pathol. Res. Pr..

[51] Chen L., Lu Q., Chen J., Feng R., Yang C. (2021). Upregulating miR-27a-3p Inhibits Cell Proliferation and Inflammation of Rheumatoid Arthritis Synovial Fibroblasts through Targeting Toll-like Receptor 5. Exp. Ther. Med..

[52] Wu J., Sun Z., Sun H., Li Y. (2018). MicroRNA-27a Promotes Tumorigenesis via Targeting AKT in Triple Negative Breast Cancer. Mol. Med. Rep..

[53] Ren Y., Fu F., Han J. (2015). MiR-27a Modulates Radiosensitivity of Triple-Negative Breast Cancer (TNBC) Cells by Targeting CDC27. Med. Sci. Monit..

[54] Zhang L.-Y., Chen Y., Jia J., Zhu X., He Y., Wu L.-M. (2019). MiR-27a Promotes EMT in Ovarian Cancer through Active Wnt/***β***-Catenin Signalling by Targeting FOXO1. Cancer Biomark.

[55] Uyanik B., Goloudina A.R., Akbarali A., Grigorash B.B., Petukhov A.V., Singhal S., Eruslanov E., Chaloyard J., Lagorgette L., Hadi T. (2021). Inhibition of the DNA Damage Response Phosphatase PPM1D Reprograms Neutrophils to Enhance Anti-Tumor Immune Responses. Nat. Commun..

[56] Kucheryavenko A.S., Muzyko E.A., Perfilova V.N., Kaplanov K.D., Frolov M.Y. (2025). The Role of the PPM1D Gene in Tumor Pathogenesis. Biomed. Khim..

[57] Zhang L., Hsu J.I., Goodell M.A. (2022). PPM1D in Solid and Hematologic Malignancies: Friend and Foe?. Mol. Cancer Res..

[58] Milosevic J., Treis D., Fransson S., Gallo-Oller G., Sveinbjörnsson B., Eissler N., Tanino K., Sakaguchi K., Martinsson T., Wickström M. (2021). PPM1D Is a Therapeutic Target in Childhood Neural Tumors. Cancers.

[59] Whitaker R.H., Cook J.G. (2021). Stress Relief Techniques: P38 MAPK Determines the Balance of Cell Cycle and Apoptosis Pathways. Biomolecules.

[60] Shi T., van Soest D.M.K., Polderman P.E., Burgering B.M.T., Dansen T.B. (2021). DNA Damage and Oxidant Stress Activate P53 through Differential Upstream Signaling Pathways. Free Radic. Biol. Med..

[61] Ghosh S., Javia A., Shetty S., Bardoliwala D., Maiti K., Banerjee S., Khopade A., Misra A., Sawant K., Bhowmick S. (2021). Triple Negative Breast Cancer and Non-Small Cell Lung Cancer: Clinical Challenges and Nano-Formulation Approaches. J. Control Release.

[62] Xu Y., Zhang H., Nguyen V.T.M., Angelopoulos N., Nunes J., Reid A., Buluwela L., Magnani L., Stebbing J., Giamas G. (2015). LMTK3 Represses Tumor Suppressor-like Genes through Chromatin Remodeling in Breast Cancer. Cell Rep..

[63] Rani A., Stebbing J., Giamas G., Murphy J. (2019). Endocrine Resistance in Hormone Receptor Positive Breast Cancer-From Mechanism to Therapy. Front. Endocrinol..

[64] Anbarasu K., Jayanthi S. (2018). Identification of Curcumin Derivatives as Human LMTK3 Inhibitors for Breast Cancer: A Docking, Dynamics, and MM/PBSA Approach. 3 Biotech.

[65] Wang X., Eichhorn P.J.A., Thiery J.P. (2023). TGF-β, EMT, and Resistance to Anti-Cancer Treatment. Semin. Cancer Biol..

[66] Luo W., Shi Q., Han M., Zhang Z., Reiter R.J., Ashrafizadeh M., Nabavi N., Sethi G., Nicot C., Mao Y. (2025). TGF-β-Driven EMT in Cancer Progression and Drug Resistance. Cytokine Growth Factor Rev..

[67] Chen M., Wu C., Fu Z., Liu S. (2022). ICAM1 Promotes Bone Metastasis via Integrin-mediated TGF-β/EMT Signaling in Triple-negative Breast Cancer. Cancer Sci..

[68] Janus P., Kuś P., Jaksik R., Vydra N., Toma-Jonik A., Gramatyka M., Kurpas M., Kimmel M., Widłak W. (2024). Transcriptional Responses to Direct and Indirect TGFB1 Stimulation in Cancerous and Noncancerous Mammary Epithelial Cells. Cell Commun. Signal.

[69] Chen S., Liu S., Ma K., Zhao L., Lin H., Shao Z. (2019). TGF-β Signaling in Intervertebral Disc Health and Disease. Osteoarthr. Cartil..

[70] Rizzotto D., Englmaier L., Villunger A. (2021). At a Crossroads to Cancer: How P53-Induced Cell Fate Decisions Secure Genome Integrity. Int. J. Mol. Sci..

[71] Janic A., Abad E., Amelio I. (2025). Decoding P53 Tumor Suppression: A Crosstalk between Genomic Stability and Epigenetic Control?. Cell Death Differ..

[72] Marvalim C., Datta A., Lee S.C. (2023). Role of P53 in Breast Cancer Progression: An Insight into P53 Targeted Therapy. Theranostics.

[73] Berke T.P., Slight S.H., Hyder S.M. (2022). Role of Reactivating Mutant P53 Protein in Suppressing Growth and Metastasis of Triple-Negative Breast Cancer. Onco. Targets Ther..

[74] Zhang Z., Hao R., Guo Q., Zhang S., Wang X. (2021). TP53 Mutation Infers a Poor Prognosis and Is Correlated to Immunocytes Infiltration in Breast Cancer. Front. Cell Dev. Biol..

[75] Wang Z., Strasser A., Kelly G.L. (2022). Should Mutant TP53 Be Targeted for Cancer Therapy?. Cell Death Differ.

[76] Agupitan A.D., Neeson P., Williams S., Howitt J., Haupt S., Haupt Y. (2020). P53: A Guardian of Immunity Becomes Its Saboteur through Mutation. Int. J. Mol. Sci..

[77] Patel S.A., Minn A.J. (2018). Combination Cancer Therapy with Immune Checkpoint Blockade: Mechanisms and Strategies. Immunity.

[78] Luo K. (2017). Signaling Cross Talk between TGF-β/Smad and Other Signaling Pathways. Cold Spring Harb. Perspect. Biol..

[79] Zhang J.-G., Xu C., Zhang L., Zhu W., Shen H., Deng H.-W. (2019). Identify Gene Expression Pattern Change at Transcriptional and Post-Transcriptional Levels. Transcription.

[80] Chandrashekar D.S., Karthikeyan S.K., Korla P.K., Patel H., Shovon A.R., Athar M., Netto G.J., Qin Z.S., Kumar S., Manne U. (2022). UALCAN: An Update to the Integrated Cancer Data Analysis Platform. Neoplasia.

[81] Chen Y., Wang X. (2020). miRDB: An Online Database for Prediction of Functional microRNA Targets. Nucleic Acids Res..

[82] Faul F., Erdfelder E., Buchner A., Lang A.-G. (2009). Statistical Power Analyses Using G*Power 3.1: Tests for Correlation and Regression Analyses. Behav. Res. Methods..

[83] Krajowy Rejestr Nowotworów (2019). Nowotwór Piersi. https://onkologia.org.pl/sites/default/files/Pier%C5%9B.pdf.

[84] Kalkulator Doboru Próby. https://www.naukowiec.org/dobor.html.

[85] Győrffy B. (2024). Integrated Analysis of Public Datasets for the Discovery and Validation of Survival-Associated Genes in Solid Tumors. Innovation.

[86] Győrffy B. (2024). Transcriptome-Level Discovery of Survival-Associated Biomarkers and Therapy Targets in Non-Small-Cell Lung Cancer. Br. J. Pharmacol..

[87] Szklarczyk D., Kirsch R., Koutrouli M., Nastou K., Mehryary F., Hachilif R., Gable A.L., Fang T., Doncheva N.T., Pyysalo S. (2023). The STRING Database in 2023: Protein-Protein Association Networks and Functional Enrichment Analyses for Any Sequenced Genome of Interest. Nucleic Acids Res..

