# Supplementary material for: Multi-Level Profiling of MAPK-Associated Genes and MicroRNAs Uncovers Regulatory Networks in Breast Cancer Subtypes
Source: Int J Mol Sci. 2025 Dec 7;26(24):11831. doi: 10.3390/ijms262411831 (PMC12732379; doi:10.3390/ijms262411831)
Supplement: Supplementary file 1 [file ijms-26-11831-s001.zip › ijms-4008166-supplementary.pdf]

Supplementary Table S1. Comparison of previously published pathway-specific analyses and the current MAPK–miRNA study performed on the same breast-cancer cohort

Study / Reference	Signaling Pathway Focus	Main Molecular Targets (mRNAs / Proteins)	miRNAs Investigated	Validation Techniques	Clinical / Survival Analyses	Data Type Reused in Present Study	Novel Elements in the Current MAPK–miRNA Manuscript
**Sirek et al., Int. J. Mol. Sci. 2024; 25:6546**	Dopaminergic system	DRD1, DRD2, DRD4, TH, MAOA, MAOB, COMT	miR-34a, miR-23c, miR-205-3p	RT-qPCR, ELISA	Subtype comparison, OS via KM-Plotter	Patient demographics, RNA source, technical platform	New KEGG-based selection of MAPK-related transcripts; integrated network and miRNA mapping distinct from dopaminergic axis
**Sirek et al., Front. Oncol. 2024; 14:1407538**	Histaminergic system	HRH1, HRH2, HRH4, HNMT, EDN1, EDNRA, HTR6, ADA, SLC23A2	miR-34a, miR-3140-5p, miR-4251, miR-1-3p, miR-382, miR-16, miR-650, miR-1275	Microarray, RT-qPCR, ELISA	Subtype distribution	Cohort and baseline clinicopathologic data	Novel focus on MAPK genes (MAP3K1, MAP2K4, PPM1D, LMTK3, TGFB1, TP53); integration of protein and survival data
**Sirek et al., Int. J. Mol.**	SMAD / TGF-β signaling	SMAD3, SMAD	miR-145, miR-155,	Microarray, RT-	Subtype-specific expressi	Cohort, control tissue, and	New interrogation of the

**Sci. 2024; 25:10088**		4, SMAD5, SMAD7, TGFBR2, INHBA	miR-15b, miR-21b	qPCR, ELISA	on comparison	raw microarray base layer	same transcriptomic dataset for 312 MAPK-related genes (KEGG hsa04010); new RT-qPCR + ELISA validation; miRNA correlation networks; STRING PPI and Kaplan–Meier OS analyses
**Current study (2025)**	MAPK signaling and MAPK–miRNA crosstalk	MAP3K1, MAP2K4, PPM1D, LMTK3, TGFB1, TP53	miR-21-3p, miR-23c, miR-27a-3p, miR-205-3p, miR-300	RT-qPCR, ELISA, STRING PPI, miRDB prediction, inverse-correlation analysis	Subtype-specific and overall-survival associations	—	First integrated multi-omics map of the MAPK–miRNA regulatory network within this cohort; newly generated validation and prognostic data

Legend: This table summarizes the scope of pathway-specific analyses conducted on a shared cohort of 405 Polish breast-cancer patients (luminal A = 130; luminal B HER2− = 100; luminal B HER2+ = 96; HER2+ = 36; TNBC = 43). Previous studies examined dopaminergic, histaminergic, and SMAD/TGF-β signaling axes. The current manuscript introduces a novel interrogation of the same transcriptomic backbone focused on MAPK-related genes, their regulatory miRNAs, and prognostic impact, with new experimental validations (RT-qPCR, ELISA) and STRING/KEGG-based interaction modeling. All shared datasets are disclosed for transparency; only MAPK-specific results are claimed as novel.

Supplementary Table S2. RFS analysis across breast cancer subtypes.

Gene	LumA		Direction	LumB HER2−		Direction	LumB HER2+		Direction	HER2+		Direction	TNBC		Direction
	HR	*p*		HR	*p*		HR	*p*		HR	*p*		HR	*p*	
MAP3K1	0.7 (0.4-1)	0.06	NS	0.9 (0.7-1.3)	0.7	NS	0.6 (0.3-1.3)	0.2	NS	0.8 (0.6-1.2)	0.3	NS	1.5 (0.9-2.5)	0.2	NS
MAP2K4	0.8 (0.6-1.1)	0.1	NS	0.7 (0.6-0.9)	0.001	Low worse	1 (0.6-1.6)	1	NS	0.9 (0.7-1.2)	0.4	NS	1.2 (0.9-1.6)	0.3	NS
PPM1D	1.5 (1-2.3)	0.04	High worse	0.9 (0.6-1.2)	0.4	NS	1.1 (0.5-2.5)	0.8	NS	0.9 (0.7-1.3)	0.6	NS	1.3 (0.7-2.1)	0.4	NS
LMTK3	0.7 (0.5-1)	0.05	NS	0.7 (0.5-0.9)	0.01	Low worse	0.8 (0.4-1.8)	0.6	NS	0.9 (0.6-1.3)	0.6	NS	1.4 (0.8-2.3)	0.3	NS
TGFB1	1.3 (1-1.6)	0.06	NS	1.3 (1-1.6)	0.02	High worse	1 (0.6-1.6)	1	NS	0.8 (0.6-1)	0.1	NS	0.9 (0.7-1.2)	0.5	NS
TP53	1 (0.8-1.3)	1	NS	0.8 (0.6-0.9)	0.01	Low worse	1.5 (0.9-2.4)	0.1	NS	0.8 (0.6-1)	0.06	NS	1 (0.8-1.4)	0.8	NS

LumA, luminal A; LumB, luminal B; HER2, human epidermal growth factor receptor 2; TNBC, triple-negative breast cancer; C, control; MAP3K1, Mitogen-Activated Protein Kinase Kinase Kinase 1; MAP2K4, Mitogen-Activated Protein Kinase Kinase 4; PPM1D, Protein Phosphatase, Mg2+/Mn2+ Dependent 1D (also known as WIP1); LMTK3, Lemur Tyrosine Kinase 3; TGFB1, Transforming Growth Factor Beta 1; TP53, Tumor Protein p53; NS, not significant.
